# Hyphenated high-resolution mass spectrometry—the “all-in-one” device in analytical toxicology?

**DOI:** 10.1007/s00216-020-03064-y

**Published:** 2020-11-28

**Authors:** Hans H. Maurer

**Affiliations:** Department of Experimental and Clinical Toxicology, Institute of Experimental and Clinical Pharmacology and Toxicology, Saarland University, 66421 Homburg (Saar), Germany

**Keywords:** High-resolution, Mass spectrometry, All-in-one device, Screening, Quantification, Metabolism, Metabolomics, Toxicology

## Abstract

This trend article reviews papers with hyphenated high-resolution mass spectrometry (HRMS) approaches applied in analytical toxicology, particularly in clinical and forensic toxicology published since 2016 and referenced in PubMed. The article focuses on the question of whether HRMS has or will become the all-in-one device in these fields as supposed by the increasing number of HRMS presentations at scientific meetings, corresponding original papers, and review articles. Typical examples for the different application fields are discussed such as targeted or untargeted drug screening, quantification, drug metabolism studies, and metabolomics approaches. Considering the reviewed papers, HRMS is currently the only technique that fulfills the criteria of an all-in-one device for the various applications needed in analytical toxicology.

Graphical abstract
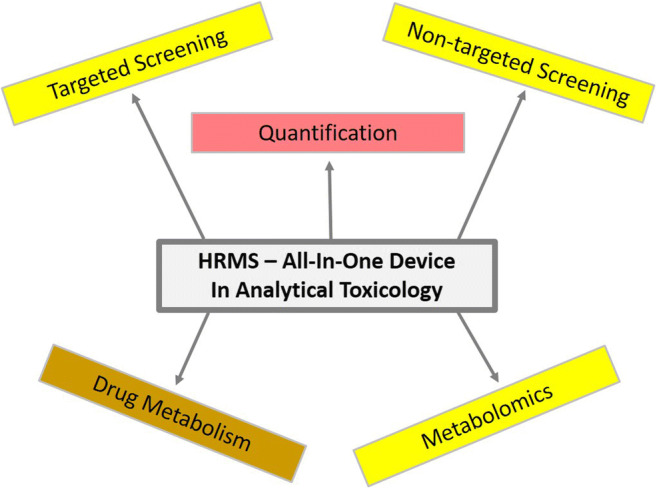

## Introduction

Since the 1980s, gas chromatography-mass spectrometry (GC-MS) has become the gold standard in analytical toxicology with selected ion monitoring (SIM) for immunoassay confirmation, targeted screening, and quantification. Full-scan monitoring providing informative and reproducible mass spectra with electron impact (EI) ionization allows comprehensive screening with a high degree of confidence using corresponding reference libraries [1–6]. In last years, the number of GC-MS papers decreased, but GC-MS with electron ionization (EI) is still in use as the backbone of the clinical and forensic laboratory [3].

Since the 1990s, liquid chromatography-mass spectrometry (LC-MS) with electrospray ionization (ESI), atmospheric pressure chemical ionization (APCI), or atmospheric pressure photoionization (APPI) revolutionized the bioanalysis also in analytical toxicology. LC coupled to tandem MS (LC-MS/MS) with selected reaction monitoring (SRM) for targeted (multi-analyte) screening and quantification or with data-dependent or data-independent product ion spectra formation for comprehensive screening has become a new gold standard [2–6].

The next trend started in the last years with the coupling of high-resolution mass spectrometry (HRMS) mostly with GC or LC for analysis of small and large molecules in analytical toxicology [2]. HRMS was developed in the 1960s with double-focusing mass spectrometers, but today time-of-flight (TOF) or Orbitrap (OT) mass analyzers are common, mostly as hybrids with triple quadrupoles (QTOF, QOT) or ion traps in front allowing fragmentation to reproducible MS/MS spectra [3–7]. The high mass resolution allows differentiation of isobaric compounds with the same nominal mass but different elemental compositions. Thus, mass traces of coeluting isobaric compounds, e.g., endogenous biomolecules, can be excluded increasing the selectivity and thus sensitivity. The elemental composition of a molecule can be calculated by accurate determination of the parent and fragment masses allowing provisional identification of unknown compounds, e.g., by comparing with lists of the exact masses and empirical formulas of potential poisons [8]. However, isomeric compounds can only be differentiated by different fragmentation [9]. The increasing number of HRMS presentations at scientific meetings, corresponding original papers, and review articles [2–7, 10–12] may lead to the presumption that HRMS has or will become the all-in-one device for targeted or non-targeted (also named untargeted) screening, quantification, drug metabolism, and metabolomics in analytical toxicology, namely in clinical and forensic toxicology, forensic chemistry, doping control, etc. Therefore, the aim of the present trend article is to confirm this presumption considering English-written papers published since 2016 and referenced in PubMed preselected with the terms “HRMS and (toxicology or forensics or doping).” Out of them, review articles covering a particular aspect of a trend and typical—exemplary—original papers supporting the corresponding trends have been selected as the number of the citations is limited for trend articles.

## Screening for detection of drugs, poisons, and/or their metabolites

In contrast to other fields of analytical chemistry, the analysis in clinical and forensic toxicology has to start with a screening for detection of often unknown drugs or poisons. Depending on the case and the clinical signs of intoxication, the screening has to cover a limited number of compounds or even over 10,000 potential poisons. Thus, the analytical strategy is different; either a targeted screening or a non-targeted comprehensive screening can be performed.

LC-MS/MS in the SRM mode was established over the last years as the standard for multi-analyte targeted screening, often combined with quantification [2, 13–15]. The identification power depends, of course, on the selectivity and the number of monitored transitions. Selectivity can markedly be improved by using HRMS doubling the identification points per selected ion [16]. Another advantage of HRMS is the option of combined targeted and untargeted screening. Thoren et al. [17] compared a typical LC-MS/MS targeted screening with a triple quadrupole linear ion trap with a non-targeted LC-HRMS/MS method with advantages for general unknown drug screening. Both methods used information-dependent acquisition of product ion spectra. LC-HRMS/MS was slightly less sensitive, but offered an open unknown screening. Further advantages will be discussed in the following.

**Fig. 1:**
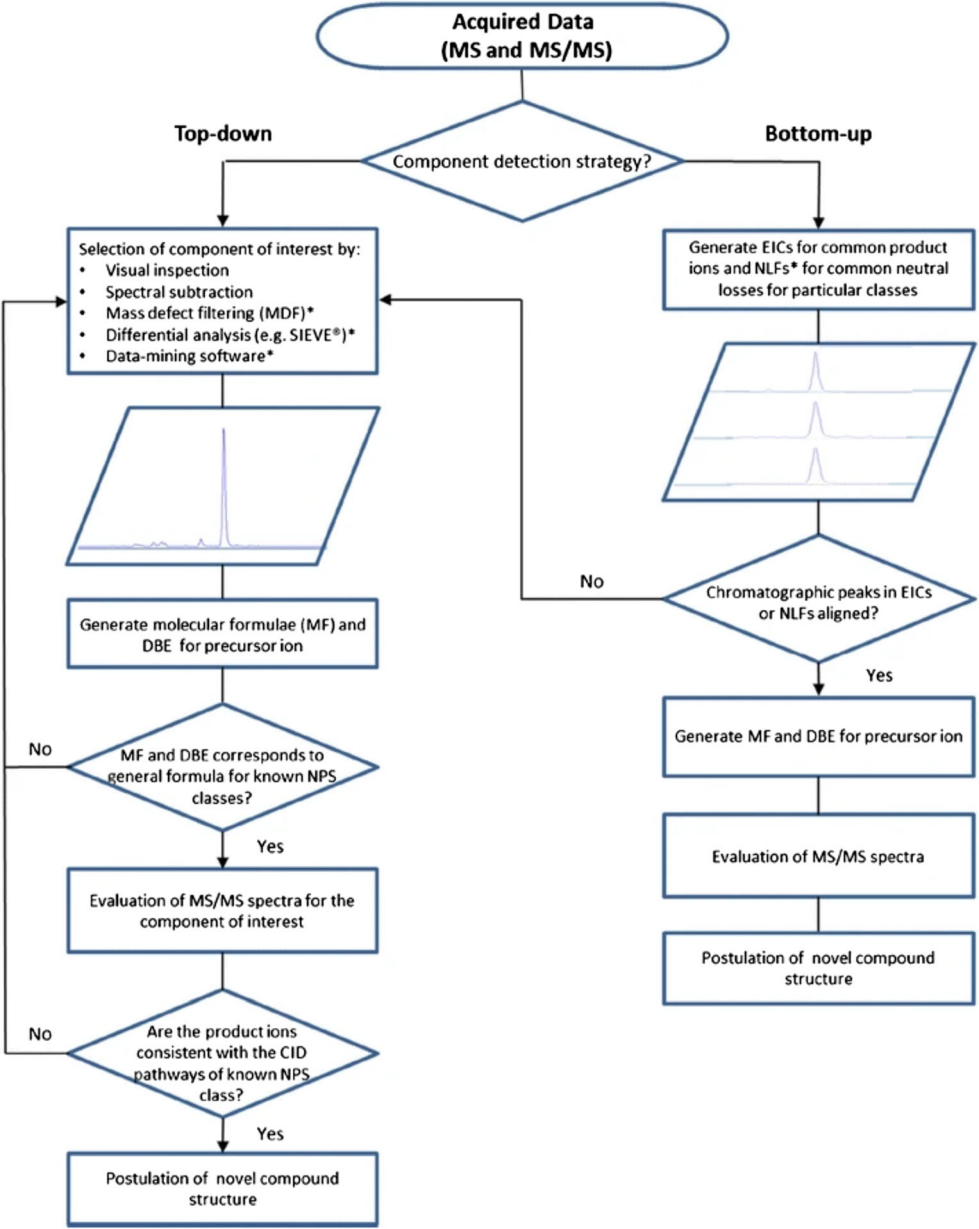
Comparison of top-down and bottom-up non-targeted screening workflows using HRMS (taken from Pasin et al., *Anal Bioanal Chem*. 2017;409:5821–36). Details in ref. [11]

Interestingly, GC coupled to HRMS (GC-HRMS) was applied for a high-throughput screening for detection of about 300 drugs and poisons in human blood using an OT analyzer [18]. However, considering the limitations of GC [9] such as risk of thermal degradation, limited volatility without derivatization, and less sensitivity, the advantage over corresponding LC-HRMS approaches cannot be assessed. In the last few years, a clear trend to highly selective and sensitive screening by LC-HRMS/MS with QTOF or QOT analyzers was observed [19–21], particularly since hundreds of so-called new psychoactive substances (NPS) appeared on the drugs of abuse market per year [2–6, 12, 22]. Pasin et al. [11] critically reviewed applications for NPS analysis and highlighted the advantage to detect and tentatively identify novel analogs without the need for certified reference materials or comprehensive mass spectral libraries. They discussed non-targeted screening strategies as a two-step process that involves the discovery or detection of a component followed by putative identification. Component discovery has been identified as the most problematic step, which can be categorized into two different approaches, top-down or bottom-up, as illustrated in Fig. 1. The current role of HRMS in NPS analysis was recently discussed with experts in this field [23]. Considering all advantages, HRMS tend to replace conventional quadrupole-based MS, particularly using integrated targeted/non-targeted screening for detection of known and new substances also with retrospective data mining [24].

In the following, selected examples for typical LC-HRMS screening approaches are discussed showing the trend of universality of this technique. In direction to compliment or even replace well-established GC-MS general unknown screenings [25–27], Helfer et al. [20] developed an LC-QOT-MS/MS standard urine screening approach in full-scan mode after positive/negative switching and data-dependent acquisition for unknowns. A compound was positively identified when the corresponding accurate mass precursor ion and the five most intense fragment ions were detected and the MS/MS spectrum fits well with the corresponding full HR-MS/MS reference library of parent drugs and their metabolites [28]. This approach was successfully transferred to blood analysis providing fast, simple, and robust screening and identification of a broad range of drugs within therapeutic ranges [21]. However, in contrast to GC-EI-MS reference libraries (e.g., ref. [29]) running with different apparatus types, LC-(HR)MS libraries (e.g., ref. [28]) can be more apparatus-depending as ionization, collision energy, MS/MS conditions, etc. may have a significant influence on the transferability, but can be limited by adopting and standardizing these parameters [30]. Partridge et al. [31] described another comprehensive LC-QTOF-MS/MS blood screening also using data-dependent acquisition, and an in-house retention time, accurate mass, and MS/MS spectral database. As advantage of such methods, they can be easily updated with new compounds without affecting method performance. Finally, an LC-QTOF-MS/MS with data-independent acquisition was developed for serum screening and applied to authentic serum and post-mortem femoral blood samples in comparison to GC-MS [32]. Not surprisingly, the HRMS method could detect much more drugs than the GC-MS approach.

Besides these general approaches, various methods were published for particular drug groups e.g., hallucinogenic phenethylamines (non-targeted) [33], low-dosed opioids (non-targeted data acquisition coupled with targeted data processing) [34], or synthetic cannabinoids (non-targeted) [35]. Thanks to its high sensitivity, LC-HRMS/MS was successfully applied also for broad-spectrum drug screening in low sample volumes such as dried blood spots [36] or in samples with low concentrations such as hair samples [37], urine after dilute-and-shoot application [38], or wastewater [39]. Finally, Mollerup et al. [40] described a new approach with LC-ion mobility-HRMS/MS for broad scope screening based on prediction of collision cross section and retention time with machine learning using artificial neural networks. Together with the exact mass, tentative identification of new compounds could be performed with in silico predicted reference values for improving confidence and filtering false-positive identifications.

Besides GC or LC coupling, paper spray ionization coupled to QOT (PSI-HR-MS/MS) allowed comprehensive urine drug screening [41]. Its screening power was compared to that of published LC-HR-MS/MS procedures [42] showing that PSI-HR-MS/MS was suitable, but limitations should be considered such as limited detection of drugs in low concentrations and risk of false-positive or false-negative results caused by mixed spectra. McKenna et al. [43] compared PSI-HR-MS/MS with conventional LC-MS/MS resulting in acceptable qualitative and quantitative agreement. A further ambient coupling was described by Duvivier et al. [44] using direct analysis in real-time HRMS for drug testing by longitudinal scan of intact locks of hair. Data-dependent product ion scanning allowed detection of various drugs of abuse in a single hair confirmed by accurate mass and fragmentation patterns. In forensic chemistry, drugs (e.g., NPS) in solid and liquid samples could be detected using ambient ionization techniques coupled to HRMS [45], namely by laser diode thermal desorption or atmospheric solids analysis probe allowing fast analysis of a wide range of samples with minimal or no sample preparation. This ambient coupling confirmed again the universality of HRMS.

## Quantification

For assessing the extent of impairment or severity of poisoning, quantification mainly in blood (plasma, serum) is needed. So far, LC-MS/MS mostly in SRM mode is the method of choice for quantification, often combined with targeted screening (see above), allowing multi-analyte approaches saving time and resources [2, 4, 6, 13, 46]. The question arises whether there is a trend that HRMS will take over also this field. Recent papers and review articles clearly indicate this, particularly for low-dosed drug or in low-volume samples [7, 11, 22]. For example, Caspar et al. [47] developed a quantitative approach for low-dosed hallucinogens and opioids in blood plasma using LC-OT-MS/MS with alternating HR full-scan and all-ions fragmentation MS. This allowed identification and quantification with no limitations on the number of monitored compounds and reevaluation of the acquired data using group-indicating fragment ions, e.g., for new or unexpected analytes. Thomas et al. [48] described simultaneous quantification of insulin, its synthetic analogs, and C-peptide in human plasma by LC-OT-MS/MS with targeted single ion monitoring experiments for the multiply protonated precursors of the target peptides or alternatively with product ion experiments for the respective five- or fourfold protonated precursors. Further procedure for insulins was recently reviewed by the same group [49] concluding that HRMS provides the sensitivity required to determine analyte concentrations in the sub-ng/mL level. Another highly sensitive approach was published [50] for determination of anticoagulant rodenticides in blood by LC-QTOF-MS/MS with parallel reaction monitoring providing the highest sensitivity. Finally, Kronstrand et al. [51] developed an LC-QTOF-MS/MS method using the all-ions mode for quantification of low concentrations of drugs in hair showing that HRMS found its way also in alternative matrix testing.

## Metabolism of drugs of abuse

Studies on drug metabolism are mandatory in drug discovery and development and toxicological risk assessment, and also for developing urine screening assays particularly for lipophilic drugs detectable often only as metabolites in urine [7]. For example, NPS are sold without any preclinical study, and thus, no or limited information about their excretion form is known. Thus, clinical and forensic toxicologists started with analytical strategies for identification of the metabolites and their formation pathways [7, 52–55] using animals or human in vivo, ex vivo, or in vitro samples such as blood, urine, primary hepatocytes, cell cultures, S9 fraction, microsomes, or cytosol. Various review articles [7, 52, 54–57] confirm that HRMS plays the major role in this field, particularly in non-targeted modes allowing retrospective data mining [56]. Again, HRMS provides the elemental composition of the parent and fragment masses allowing to identify the type of metabolic changes and in most cases the position in a particular part of the molecule, but not the exact position, e.g., in an aromatic ring system [7]. However, the latter is of minor relevance in developing urine screening assays.

## Metabolomics techniques in analytical toxicology

Since the last few years, metabolomics plays also a role in clinical and forensic toxicology and doping control. Besides conventional GC- or LC-MS methods, LC-HRMS was established particularly for untargeted metabolomics studies, again because of its high specificity, sensitivity, and flexibility [58–63]. There are two main application fields, one focusing on the change of the endogenous compounds under the influence of drug administration [62, 64–69] or sample manipulation [65, 70–74] and one on the use of metabolomics techniques for investigating the metabolism of new drugs, namely of NPS [60, 61, 75]. Metabolomics could also play a role in doping control, e.g., for detecting hormone abuse considering that hormones have a strong influence on human endogenous metabolism changing several endogenous parameters [76].

## Outlook

The papers reviewed in this article clearly show that HRMS is currently the most powerful and flexible technique in analytical toxicology used for various applications such as targeted and non-targeted screening, quantification, drug metabolism, and metabolomics. Of course, also for HRMS, potential pitfalls have to be considered and details can be found in ref. [9]. Today, HRMS is the only technique that fulfills the criteria of an all-in-one device for the various applications needed in analytical toxicology. It can be expected that HRMS will become the gold standard and that its application will replace most of the assays with other techniques in future, of course considering suitable separation and/or ionization techniques such as GC with EI or LC with ESI, APCI, or APPI. Current limitations of HRMS techniques are the comparably expensive apparatus and the need of well-skilled operators. Another problem is the enormous size of (full scan) data requiring huge storage and fast and sophisticated software for data evaluation. Although over time the costs are becoming lower and the software packages have improved, the costs still limit the widespread distribution in routine laboratories and the software needs to become more user-friendly.
